# Deep learning-level melanoma detection by interpretable machine learning and imaging biomarker cues

**DOI:** 10.1117/1.JBO.25.11.112906

**Published:** 2020-11-27

**Authors:** Daniel S. Gareau, James Browning, Joel Correa Da Rosa, Mayte Suarez-Farinas, Samantha Lish, Amanda M. Zong, Benjamin Firester, Charles Vrattos, Yael Renert-Yuval, Mauricio Gamboa, María G. Vallone, Zamira F. Barragán-Estudillo, Alejandra L. Tamez-Peña, Javier Montoya, Miriam A. Jesús-Silva, Cristina Carrera, Josep Malvehy, Susana Puig, Ashfaq Marghoob, John A. Carucci, James G. Krueger

**Affiliations:** a The Rockefeller University, Laboratory of Investigative Dermatology, New York, New York, United States; b Icahn School of Medicine at Mount Sinai Medical Center, Department of Dermatology, New York, New York, United States; c Hospital Clínic de Barcelona, Universitat de Barcelona, Department of Dermatology, Barcelona, Spain; d Hospital Alemán, Department of Dermatology, Buenos Aires, Argentina; e Universidad Nacional Autónoma de México, Dermato-Oncology Clinic, Research Division, Faculty of Medicine, Mexico City, Mexico; f Universidad San Sebastian, School of Medicine, Concepción, Chile; g Institut d’Investigacions Biomediques August Pi I Sunyer, Barcelona, Spain; h Instituto de Salud Carlos III, CIBER on Rare Disease, Barcelona, Spain; i Memorial Sloan Kettering Cancer Center, Dermatology Service, New York, New York, United States; j New York University, Ronald O. Pearlman Department of Dermatology, New York, New York, United States

**Keywords:** skin cancer classification, machine learning, imaging biomarkers, sensory cue integration, diagnostic application

## Abstract

**Significance:** Melanoma is a deadly cancer that physicians struggle to diagnose early because they lack the knowledge to differentiate benign from malignant lesions. Deep machine learning approaches to image analysis offer promise but lack the transparency to be widely adopted as stand-alone diagnostics.

**Aim:** We aimed to create a transparent machine learning technology (i.e., not deep learning) to discriminate melanomas from nevi in dermoscopy images and an interface for sensory cue integration.

**Approach:** Imaging biomarker cues (IBCs) fed ensemble machine learning classifier (Eclass) training while raw images fed deep learning classifier training. We compared the areas under the diagnostic receiver operator curves.

**Results:** Our interpretable machine learning algorithm outperformed the leading deep-learning approach 75% of the time. The user interface displayed only the diagnostic imaging biomarkers as IBCs.

**Conclusions:** From a translational perspective, Eclass is better than convolutional machine learning diagnosis in that physicians can embrace it faster than black box outputs. Imaging biomarkers cues may be used during sensory cue integration in clinical screening. Our method may be applied to other image-based diagnostic analyses, including pathology and radiology.

## 1 Introduction

Melanoma is the most dangerous skin cancer and the leading cause of death from skin disease. There are over 96,000 new cases in the USA annually with nearly 10,000 deaths attributed to melanoma. Worldwide, annual melanoma mortality is over 60,000 people. Gaps exist in our ability to diagnose melanomas versus nevi. Gaps also exist in providing specialty care for patients with suspicious lesions and for increasing the numbers of patients seeking potentially life-saving melanoma diagnosis from primary care providers. Despite the fact that many patients with skin lesions first present to their primary care physicians, these physicians often lack the knowledge to differentiate benign from malignant lesions. Despite evidence that early detection increases survival, and despite the need for technology to enhance screening to an expert level on a wide scale, there is uncertainty regarding the effectiveness of state-of-the-art technological methodology in clinical dermatologist screening.^1^ Improved screening may prevent melanoma deaths (7230 in the USA in 2019^2^) while decreasing unnecessary invasive procedures because screening guides the binary decision for or against surgical biopsy. Technology can translate down the expertise hierarchy structure of clinical practitioners to enhance diagnosis in less-specialized medical practices. Table 1 shows that for each American to be evaluated by an expert dermoscopist, the experts would need to see millions of patients, which is not feasible, whereas if technology could enable common providers, such as general practitioners to screen with expert precision, the screening diagnostic net could be extended because providers would screen hundreds (not millions) of patients. Recent advances in machine learning have shown promise for high-performing computational diagnostic tools in multiple clinical areas,^3^^,^^4^ but the noninterpretability of data-intensive deep learning models remains a barrier to widespread deployment. Because the skin is accessible to relatively inexpensive and noninvasive diagnostic imaging, and because clinicians rely heavily on visual inspection, melanoma is an ideal case study.

**Table 1:** The Americans/Provider ratio illustrates that there are not enough Top Dermatologists with expert dermoscopy training to evaluate the entire US population. All dermatologists (20,000 in USA) automatically qualify as nonexpert dermoscopist screeners with board certification. An unmet healthcare need is to address the screening accuracy gap between top dermatologists and the broader medical network. Eclass potentially translates the top dermatologists’ pattern recognition skills (and associated diagnostic precision) to general dermatology and the broader community of nonexpert screeners.

Provider type	US population per provider
Top dermoscopists	6,480,000
Dermatologists	32,400
In-pharmacy evaluation	4830
General practitioner	379

Melanoma growth rate is variable and multifactorial. Screening is typically done with dermoscopy using a dermatoscope, which is a low-magnification, illuminated, polarized^5^ contact imaging device widely used in dermatology practice.^6^ A recent observational study^7^ regarding the rate of growth in vertical and radial growth phase of superficially spreading melanomas found that melanomas in the vertical growth phase invade by 0.13±0.16  mm/month. A delay in detection of a few months can negatively impact prognosis since growth beneath the ∼0.3-mm-deep basal layer basement membrane constitutes invasion toward metastasis. It would be a significant medical advancement if diagnostic imaging technology could improve early detection with machine learning and associated automated image processing devices to predict underlying pathology from noninvasive dermoscopy images. Yet careful consideration of biases in training data suggests that there are ethical concerns, such as the fact that training data does not always represent all skin types. Convolutional neural networks (CNN)^8^ may be inappropriate for stand-alone diagnostic medical decision making because physicians cannot have confidence in a computer-derived diagnostic risk score without understanding the underlying computational diagnostic process.

**Fig. 1:**
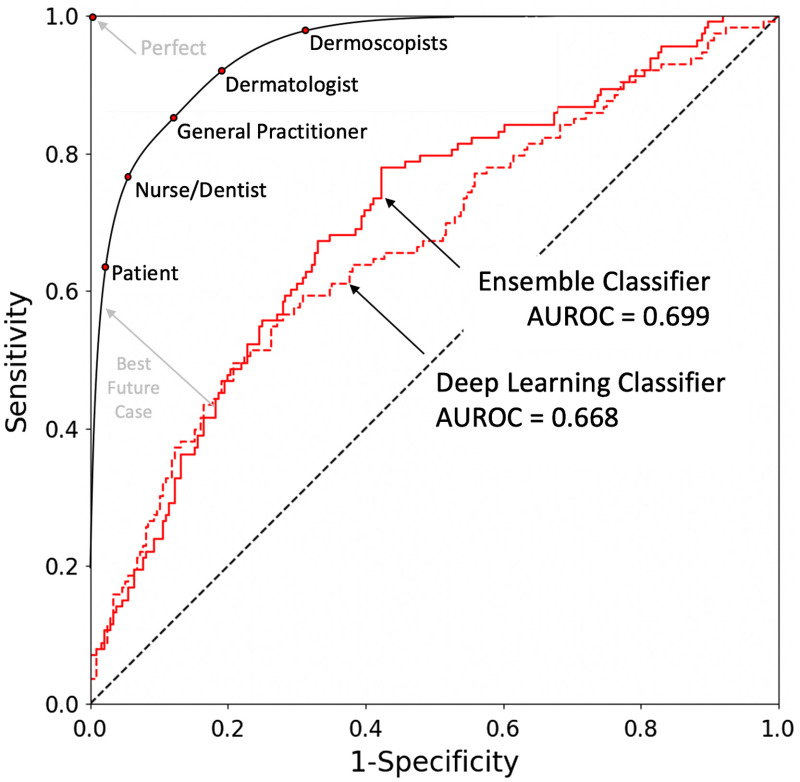
Receiver-operator characteristic curve for the convolutional neural network (CNN) versus the ensemble classifier (Eclass). In this comparison trial run, as in the case of 75% of our trial runs, Eclass out-performed CNN, with a greater area under the receiver-operator characteristic curve (AUROC). Although Eclass outperformed CNN in this study, both the Eclass and CNN predictive models are expected to improve with larger training data sets. A theoretical curve, with minimum AUROC diagnostic performance for translation, shows various screeners, susceptible to using the technology at different parametric ROC curve values (red dots). These range from the patient—whose high-specificity App would more accurately diagnose benign lesions that do not require escalation—to trained professional dermoscopists, who would value seeing the imaging biomarker cues in a high-sensitivity App that helps them be sure they aren’t missing rare or difficult-type lesions.

**Fig. 2:**
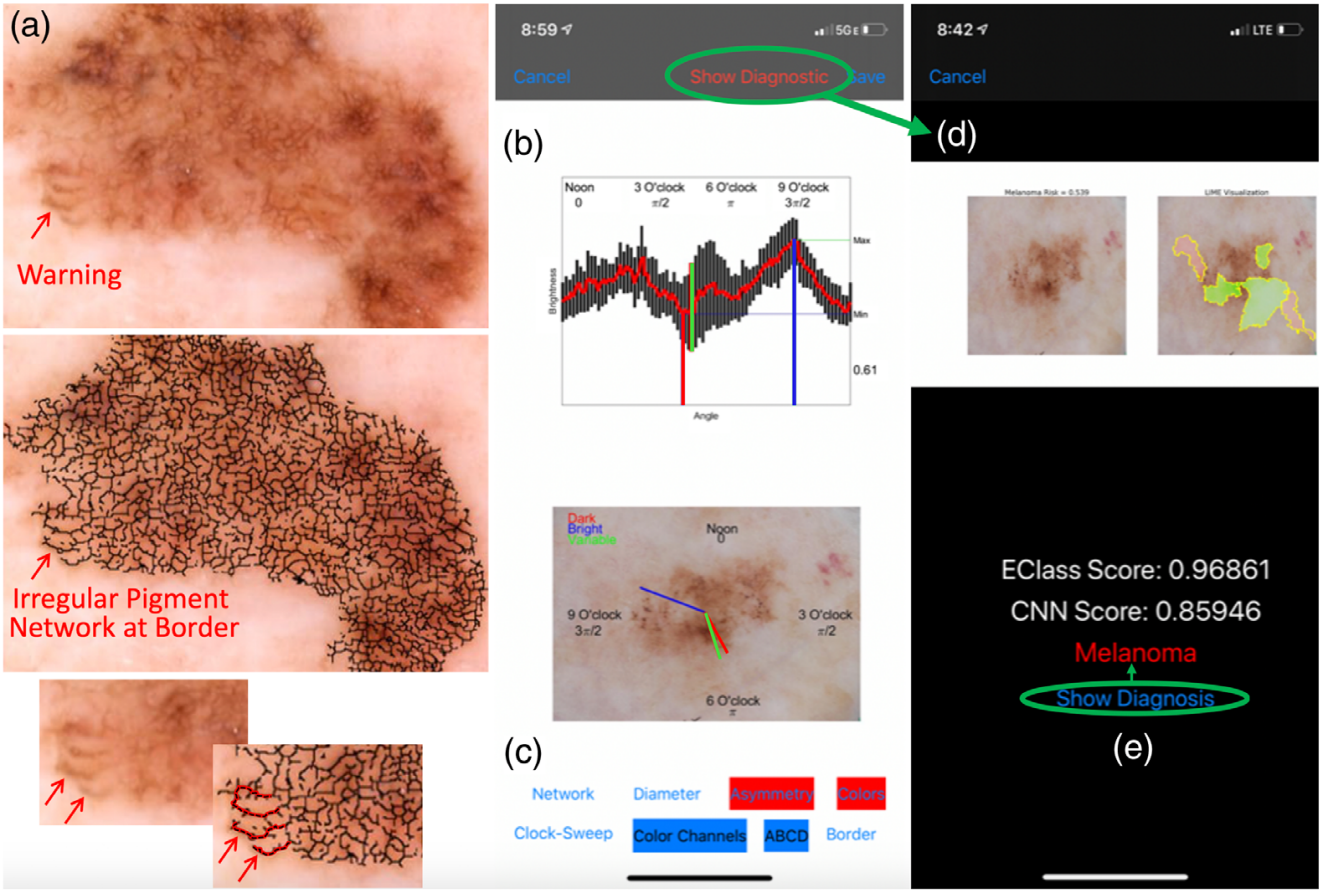
Example visualization of melanoma imaging biomarkers and machine learning diagnostic App “Eclass Imaging Biomarkers” downloaded from Mac App Store. (a) Identification of abnormally long finger-like projections in the pigmented network at the peripheral border of the lesion. (b), (c) Screen captures from the Eclass Imaging Biomarkers, freely available at Mac App Store; (b) shows a radial brightness analysis as an example of a searchable imaging biomarker set (c) where the imaging biomarkers highlighted in red (Asymmetry and Colors) indicate quantitative values that fall on the statistically malignant range of the spectrum. Since no imaging biomarkers are highlighted green, users know that the algorithm found no normal features. Green circles connected to arrows indicate analytic flow with the App. Clicking on “Show Diagnostic” (b) leads to the (d) diagnostics screen, where clicking on “Show Diagnosis” reveals the standard histopathological diagnosis. This clinical diagnostic workflow from dermoscopy image (a) to imaging biomarkers (a–b) and to Eclass and CNN scores (d) could inform screening of new images acquired in the by enabling clinicians to access automated digital diagnostics.

There exists an unmet need to better understand diagnostic processes utilized by machine learning-derived tools. As a more interpretable alternative^9^ to CNN, we considered a diagnostic ensemble classifier (Eclass)^10^ of traditional (i.e., “nondeep”) machine learning approaches. It used a set of imaging biomarkers that quantify medically relevant features rather than brute force pixel analysis to freely choose salient features. Because it is “transparent” in that imaging biomarkers are visual features, implementation of Eclass could result in more medical accountability and confidence than CNN. Herein, our context was melanoma detection, but digital imaging biomarkers based on visual sensory cues can be applied to any image-based diagnostic analysis including pathology and radiology. Figure 1 shows a sample run for each machine learning method and Fig. 2 shows a graphic user interface application (App) capable of displaying imaging biomarker cues (IBCs) to illustrate clinical and pathological features, aiding in visual interpretation of imaging diagnoses.

## 2 Methods

### 2.1 Imaging

Dermoscopy is a mainstream clinical imaging method for melanoma screening used in dermatology practice. This report analyzes two different cohorts of demoscopy images in different countries using different dermatoscope imaging systems. The first cohort^10^ consisted of alcohol-coupled, nonpolarized dermoscopy images of primary melanoma skin cancers versus abnormal nevi acquired with the EpiFlash™ (Canfield Inc., New Jersey) dermatoscope attached to a Nikon D80 camera, where each image contained 1 to 5 megapixels after cropping in New York. The second cohort, presented for the first time here, consisted of digital dermoscopy images acquired with the Dermlite Foto (3Gen Inc., San Juan Capistrano, California), sized 5.9±2.7 (mean ± standard deviation) megapixels depending on lesion size in Barcelona, Spain. All lesions were pigmented lesions that did not demonstrate a benign pattern^11^ under dermoscopy. The current data set of 668 images was reduced to 349 images (one lesion per patient and one lesion per image) by filtering out images with hair or surgical ink markings,^12^ lesion borders that extend beyond the image field of view, or other features that prevented imaging biomarker computation, like cases with extreme atypia such as those that were ulcerated, nodular/palpable, or did not fit within the field of view of the dermatoscope were excluded. Both the CNN classifier^3^ and Eclass classifier^10^ were trained on the same set of 668 images (113 melanomas and 236 nevi) and the diagnostic performances of the resulting models were compared. CNN is a leading deep learning approach while Eclass is our approach that implements a “wisdom of the crowd” approach of sampling the prediction of a broad range of machine learning predictive models. Eclass has no convolutional aspect so it has the benefit of being more easily interpretable by clinicians.

### 2.2 Eclass and Imaging Biomarkers Versus Deep Learning

**Fig. 3:**
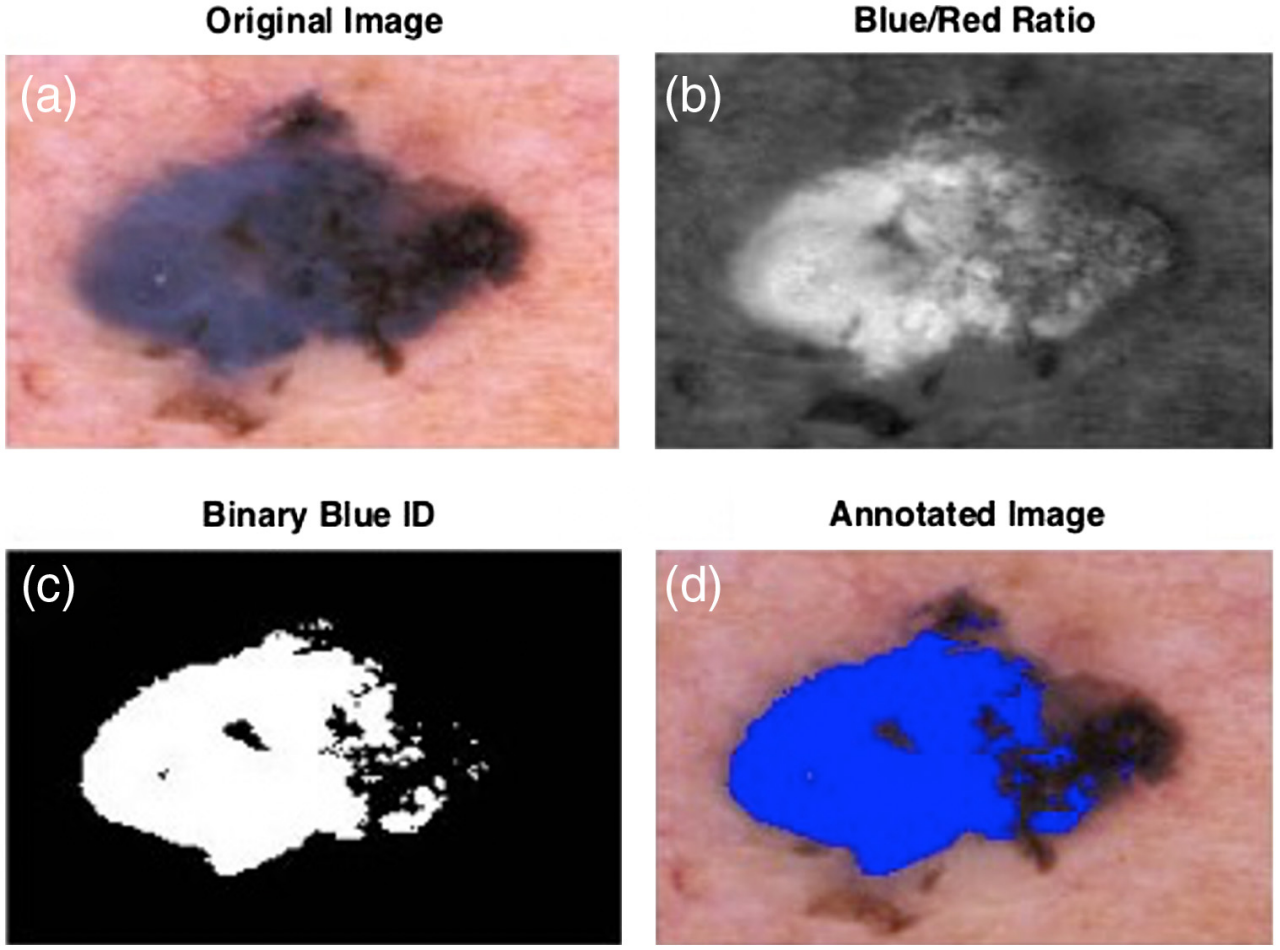
Blue gray color (a simple imaging biomarker). Referred to as a “blue-white veil” in dermoscopy, this manifestation of the Tindal effect is a statistically-significant melanoma discriminant. The lesion (a) is a melanoma. The ratio of the blue pixel intensity to the red pixel intensity (b) is the first of three steps to quantitative visualization. A simple automatic (Otsu’s method) threshold (c) is a second step to annotate the dermoscopy image with an overlay of a binary presence of a blue color (d). Displaying this image to a medical professional as a visual sensory cue can augment the cognitive process of visual sensory cue integration during melanoma screening.

The CNN used the raw pixels in the images as input features whereas Eclass operated on the set of 38 imaging biomarkers, which were engineered to automatically quantify visual features that dermatologists use during sensory cue integration in manual inspection of suspicious legions. Imaging biomarkers were either binary, such as the presence [0 1] of a blue or gray dermoscopic color (Fig. 3), integers such as the number of dermoscopic colors present [0–6], or continuous such as the variation coefficient of branch length in pigmented networks, but all imaging biomarkers were numbers that were high for melanoma and low for nevus. The detailed mathematical formulas for the imaging biomarkers used in Eclass can be found in the Appendix and in Sec. S5 of the Supplementary Material^13^ of our previous publication^10^ and have been adapted and reproduced in the methods section below. Both CNN and Eclass models predicted a melanoma probability (between 0 and 1) of the invasive histopathological diagnoses for each skin lesion using the noninvasive dermoscopy image (for CNN) or the imaging biomarkers derived from that image (for Eclass). But only Eclass involved dimensional reduction to intuitive, visual IBCs.

**Fig. 4:**
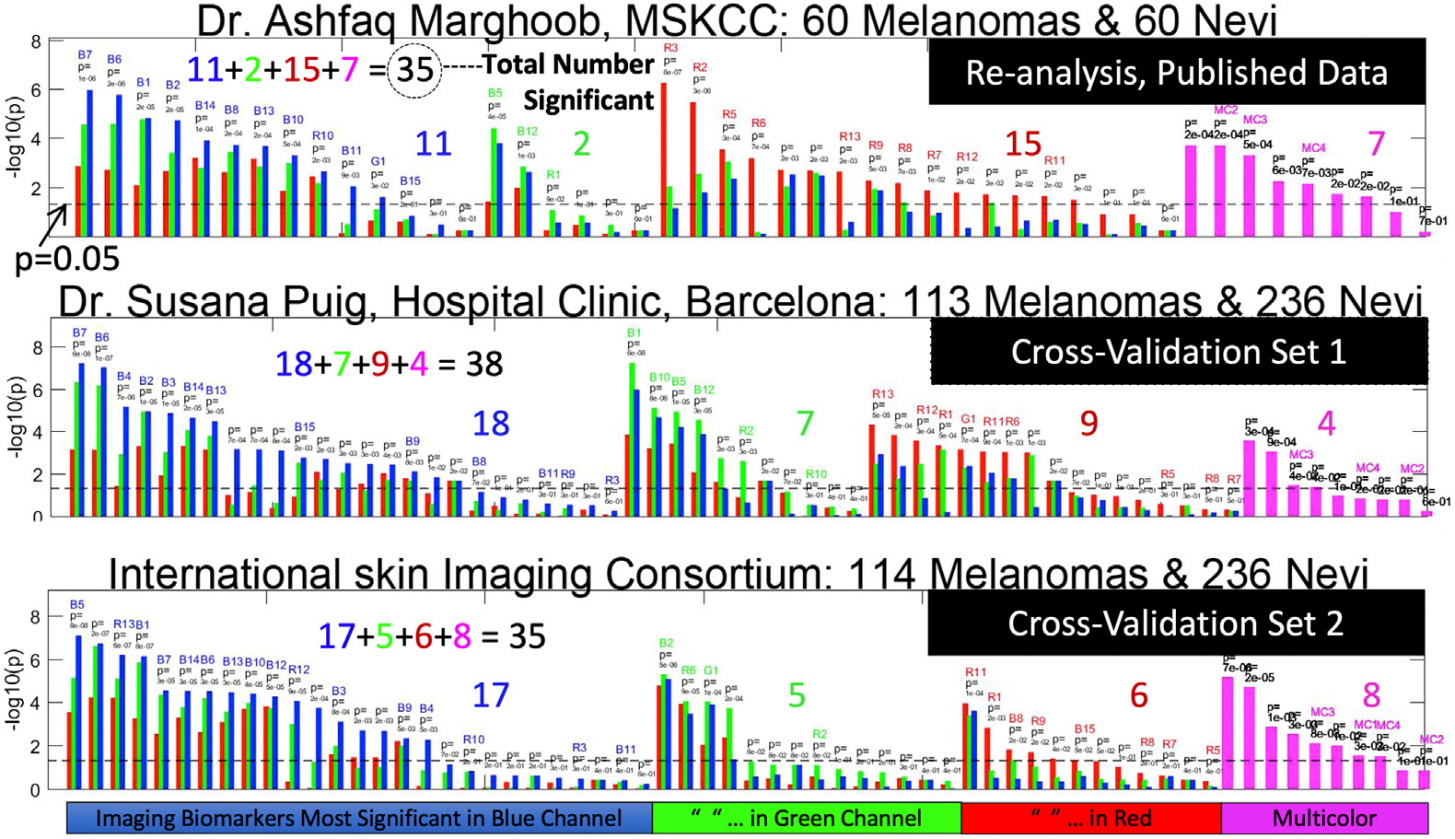
Updated imaging biomarkers statistical significance in published data versus cross-validation data sets. In this imaging biomarker re-analysis of our published data and unpublished cross validation studies, the height of the vertical bars represents the diagnostic significance of the imaging biomarkers in discriminating melanomas from nevi. Magenta bars are spectral imaging biomarkers (ones that are derived from all color channels simultaneously) and red-green-blue bars are gray-scale imaging biomarkers evaluated in the respective color channels. The imaging biomarkers are sorted by discriminant p-value between melanoma and nevus. They are categorized as those most significant in the blue channel (left), the green channel, the red channel and full spectral (right). The total number significant (p<0.05) is printed in black and the sum is tabulated for each data set (color coded numbers). The colored imaging biomarker code (eg. B7 on top left) on top of each bar, for each imaging biomarker references the written description, the mathematical derivation. This data shows that the imaging biomarkers are diagnostic across multiple screening sites because there are a similar number of significant biomarkers in cross validation sets 1 and 2 as there are in the original published data set (35, 38 and 35, respectively).

### 2.3 Statistical Methods: IBC Combination to Form the Melanoma Eclass Score

Both CNN and Eclass models were trained to predict a melanoma probability (between 0 and 1) of the invasive histopathological diagnoses for each skin lesion using the noninvasive dermoscopy image (for CNN) or the imaging biomarkers derived from that image (for Eclass). But only Eclass involved dimensional reduction to intuitive, visual IBCs described mathematically above. Eclass was trained and cross-validated within a Monte Carlo simulation as previously described in Sec. S3 in the Supplementary Materials^13^ of our previous publication^10^ and as discussed below, included a hold-out test set for each Monte Carlo iteration that was not used for training. Briefly, the 38 IBCs achieving diagnostic statistical significance (p<0.05), 4 multicolor (MC), and 34 single-color (SC) (using the most significant RGB color channel version), were input into the 12 statistical/machine learning algorithms as predictive informatics programs given in Table 2. We selected the SC IBC from the best color channel when there was any color channel for an IBC that achieved statistical significance, including that SC IBC, and also including any significant MC IBC.

**Table 2:** Broad universe of classification algorithms applied to melanoma discrimination.

Method	Description
NNET	Feed-forward neural networks with a single hidden layer^14^
SVM (linear and radial)	Support vector machines^15^^,^^16^
GLM	Logistic regression within the framework of generalized linear models^17^
GLMnet	Logistic regression with the elastic-net penalization^18^
GLMboost	Logistic regression with gradient boosting^19^
RF	Random forests^20^
RP	Classification and regression trees (CART) algorithm for classification problems^21^
KNN	K-nearest neighbors algorithm developed for classification^22^
MARS	Multiple adaptive regression splines^23^
C50	C5.0 decision tree algorithm for classification problems^24^
PLS	Partial least squares^25^
LDA	Linear discriminant analysis^26^

The statistical classification methods for machine learning were chosen to represent the broad universe of base classifiers.^27^ The C5.0 decision tree prioritized a subset of the IBCs and conducted a series of comparisons between these IBCs and a set of thresholds ultimately leading to a classification of each lesion. Each method output the melanoma likelihood for each lesion. The likelihoods produced by all methods were combined into the overall endpoint of the analysis, the ultimate best estimate of melanoma probability between zero and one, and the melanoma Eclass score. Thus, we built a predictive model that combined the IBCs into a risk score for probability of melanoma.

Our framework was set first to identify the most discriminative IBCs upon which the predictive model will be built. To this end, we first evaluate the differences between melanoma and nevus for each one of the seven multicolor IBCs and also for the RGB channel-specific IBCs (41 IBCs for each red, green, and blue channels). For this univariate assessments, two-sided unpaired t-tests, Wilcoxon–Mann–Whitney, and chi-square tests were used for continuous (e.g., IBC B1), ordinal (e.g., IBC MC1), and categorical (e.g., IBC MC4) IBCs, respectively. Of the total 130 IBCs evaluated in our current data set (Fig. 4, Cross-Validation Set 1), 38 (4 multicolor, 9 red, 7 green, and 18 blue IBCs) were selected as the most significant discriminators (p<0.05) between melanoma and nevi to continue to the multivariate discrimination stage. When significant differences for a given IBC were found in more than one channel, the most discriminative channel regarding its p-value was selected. This set of 38 discriminative IBCs measured from all the 349 lesions were used as inputs for our predictive model.

**Fig. 5:**
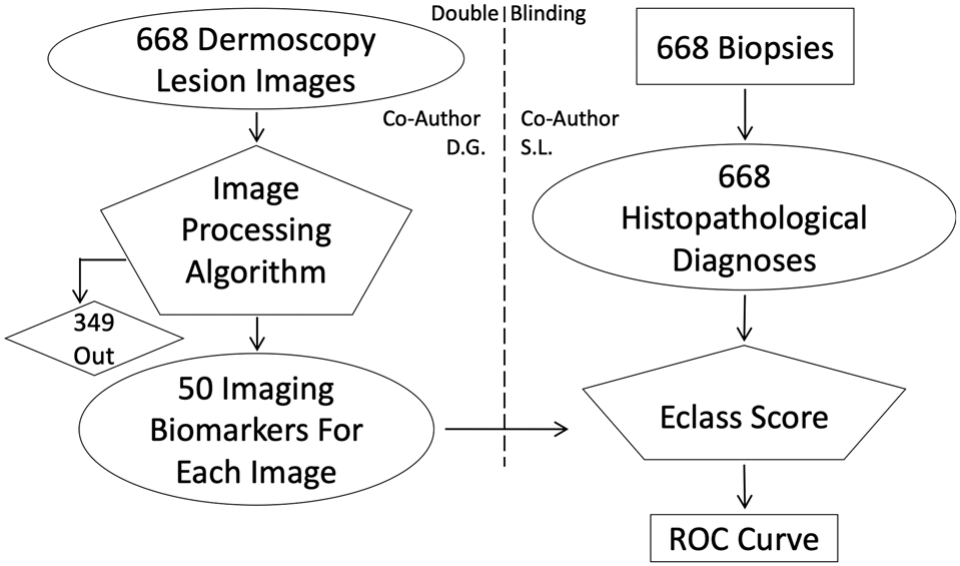
This double-blinded study retrospectively tested prediction of the histopathological biopsy diagnosis using only the dermoscopy images acquired just prior to biopsy. Co-author S. L. assembled and distributed the latter to Co-author D. G. and the former as an input to the Eclass algorithm.

**Fig. 6:**
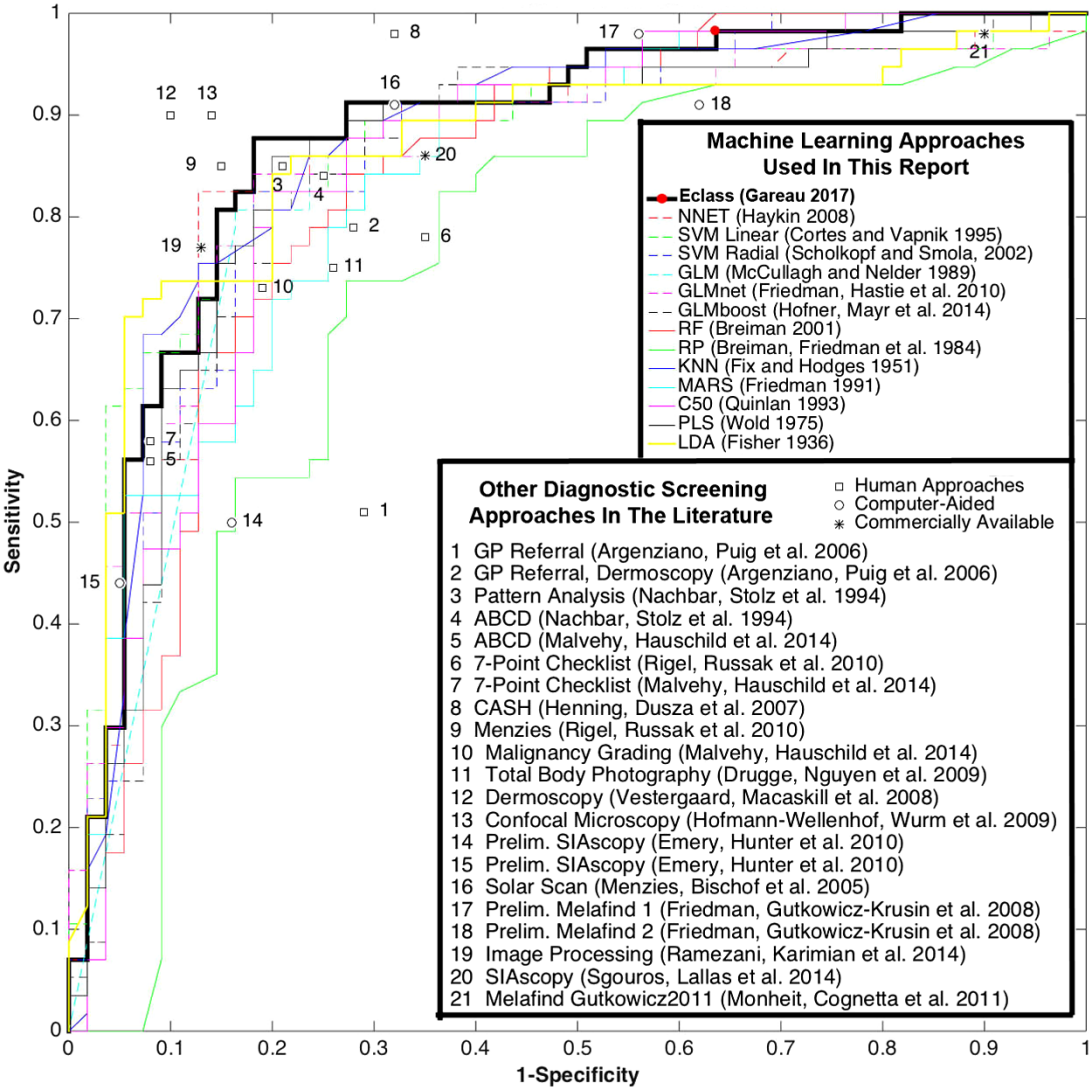
Diagnostic performance results vs. published techniques. The receiver-operator characteristic (ROC) curves for the individual machine learning approaches (thin colored lines) are outperformed by the compound Eclass score (thick black line) which is the median of the individual risk scores. Literature data points are marked with symbols that indicate if they are mostly human (square) or computer (circle)-derived.

Figure 4 compares our current results (validation set 1) to the imaging biomarkers computed on the data set from our previous publication. As such, Fig. 4 (Top) can be compared to Fig. 2 in the previous publication,^10^ to show that our algorithm has been minorly updated to achieve slightly better performance on the previously published data than previously published, with two more imaging biomarkers achieving statistical significance than before and incremental increase in area under the receiver operator characteristic curve (AUROC). Figure 4 arranges the imaging biomarkers in their order of statistical significance in discrimination between melanomas and nevi that are clinically dysplastic. The middle figure, on data from the clinic of Dr. Puig, is the data reported in this publication. Above each imaging biomarker’s significance is an alphanumeric code that correlates the imaging biomarker to its mathematical derivation below. More imaging biomarkers were statistically significant in the blue channel in cross-validation set 1, due to factors such as the differences in imaging systems and patient populations. The imaging biomarker that was the most significant blue channel imaging biomarker (B1) in our original publication had greater significance in the green channel in the current study. The meaning of imaging biomarkers that do not have a label is that these imaging biomarkers were not statistically significant in our original study (p<0.05) but are in the current study. The description of the statistical methods of Eclass has been modified from the methods in the Supplementary Material^13^ of our original publication^10^ to reflect the statistical significance breakdown in the current study. The data in Fig. 4 show that the imaging biomarkers behave similarly across data sets and that a similar number of imaging biomarkers are statistically significant is similar across study sets.

### 2.4 Clinical Study -to- Predictive Model

Figure 5 shows the overall study design by which we build a model with our experimental data including paired dermoscopy and histopathological diagnoses. The double-blind study attempted prediction of the biopsy-based histological diagnosis (melanoma or atypical nevus) using only the pre-biopsy dermoscopy image and the IBCs derived from it.

**Fig. 7:**
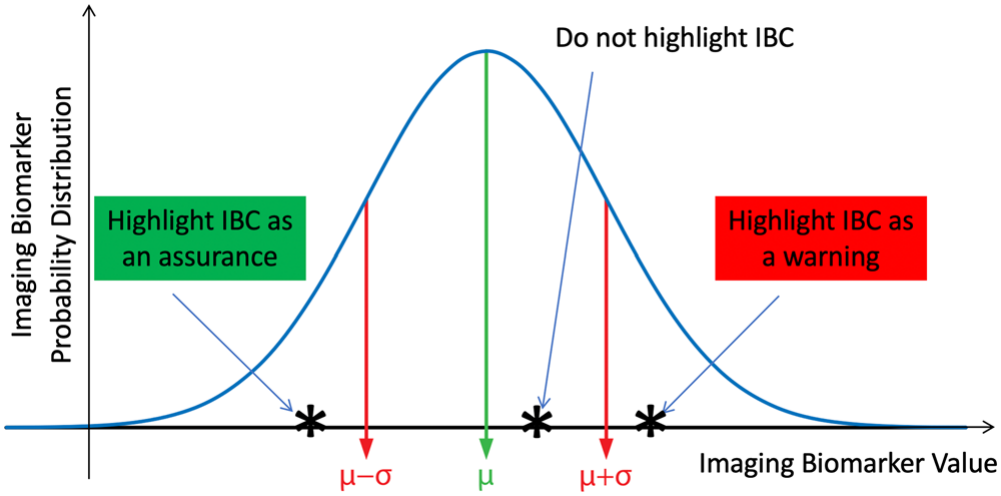
GUI/APP minimalist IBC visualization strategy. We designed IBC visualization methodology such that the display reproduced the dermoscopic image with a visual representation of the most important IBCs used by the predictive models in generating a melanoma risk score. When IBCs are less than a standard deviation below their mean (across a population of lesions), their names are visualized with green background as an assurance whereas if they are more than a standard deviation above the mean value, they are visualized with red background as a warning. Examples of warnings include the Asymmetry IBC and the number of Colors IBC in Fig. 2(c).

After dermoscopy imaging and surgical excision of the imaged lesion, the standard diagnostic method of histopathological evaluation was carried out as part of routine clinical care to yield a diagnosis (melanoma or nevus) for each lesion in the study cohort.

The 668 binary diagnoses along with 668 correlating dermoscopic images comprised the study data. No information about the patient’s age, sex, state of sun damage, or anatomical location of the lesion was used. Dermoscopy images were randomized and coded to remove all patient identifiers, then injected into the blind study arm that generated the image-processing algorithm targeting melanoma features by extracting diagnostic IBCs without knowledge of the histopathological diagnosis.

IBCs fed a collection of 12 classification methods that range from simple to sophisticated and altogether cover different data structures. The collection of classification algorithms is given in Table 2 and includes the K-nearest neighbors (KNN),^22^ a simple and efficient nonparametric distance-based method that has been successfully applied for more than 60 years. Artificial neural networks^14^ and support vector machines (SVM)^15^^,^^16^ were included to represent high-dimensional complex nonlinear functions. To accommodate complex interactions between predictors, we incorporated four methods following the decision tree/recursive partitioning paradigm: classification and regression trees (CART),^21^ C5.0,^24^ multiple adaptive regression splines (MARS),^23^ and random forests (RF).^20^ Logistic regression^18^ and linear discriminant analysis (LDA)^26^ are based on solid statistical foundations. The former permits inference upon parameters by a probabilistic approach and the latter is one of the oldest techniques for dimensionality reduction. Partial least squares regression^25^^,^^28^ is of more recent development and simultaneously performs regression/classification and dimensionality reduction.

### 2.5 Model Estimation/Training

To estimate each of the classifiers’ parameters and to evaluate the distribution of the prediction error empirically, we created a Monte Carlo experiment. During each training iteration, the set of lesions was randomly partitioned into training (75%) and test (25%) sets. For each classifier, model parameters were estimated by maximizing a partial area under the ROC curve obtained by limiting the specificity to be within the range 0% to 40% and tuning parameters were estimated by 10-fold Cross-Validation. The best configuration for each classifier was used to predict the 25% hold-out lesions in the test set.

Ensemble of predictive algorithms likely generate more accurate predictions than single algorithms.^29^^,^^30^ The melanoma Eclass score is a diagnostic for melanoma discrimination obtained by evaluating the median probability across K available classifiers

$$\text{Eclass Score}=\text{median}\{{\mathrm{Prob}}_{i}(\text{Melanoma}|M)\};\;i=1,2,\ldots ,k,$$

where ${\mathrm{Prob}}_{i}\in \{0,1\}$ is the probability of the lesion being a melanoma, as predicted by the i’th classifier based on a set of IBCs M. Monte Carlo simulations obtain the empirical distribution of the Eclass score for each lesion. The Eclass score distribution shows that the number of false-positives (melanomas classified as nevi) is lower than the false-negatives; indicating that our classification strategy is more sensitive than specific.

### 2.6 Convolutional Neural Network

The CNN was based on a widely used ResNet-50 architecture instantiated with weights pretrained on the ImageNet database for transfer learning and modified with output layers designed for binary classification. Image augmentation (flip, zoom, and rotate) and minority class (melanoma) oversampling was used during CNN training to prevent overfitting to the training data and predictive bias toward the majority class (nevus) respectively. Pixel values for training and test images were normalized to have zero mean and standard deviation of 1. During oversampling, augmented versions of minority class images were overrepresented in the training data such that the model was trained on an equivalent number of melanoma and nevi images. Test time augmentation was used during inference wherein class predictions were generated for five randomly augmented versions of each test image and the majority vote was used as the final predicted class. The CNN model trained until validation data set accuracy had not improved for ten epochs and the resulting model, with highest validation accuracy, was saved.

### 2.7 Performance Analysis

The receiver operator characteristic (ROC) curve and the area under the curve (AUROC) were used to evaluate diagnostic performance. Each method (CNN and Eclass) produced a distribution of scores from melanoma images and a second distribution of scores for benign images and we swept the diagnosis criterion across those two distributions, plotting proportion of hits as a function of proportion of false alarm (i.e., the ROC) and calculated the area under the curve. Different cross-validation runs (10 for CNN and 1000 for Eclass) were used to generate a distribution of AUROCs for each method. We compared the methods by comparing a randomly drawn AUROCs from each method’s AUROC distribution and repeating that process to determine what percentage of the time Eclass outperformed CNN.

The visualization potential of Eclass is developed as an App for imaging biomarker visual sensory cue integration^31^ that was developed based on the results of our institutional review board (IRB)-approved human subjects research (RU DGA-0923) on clinicians using the App. Our human subjects research was used to collect data regarding specific features that the clinicians found useful and whether they were likely to implement such a technology if it was available.

## 3 Results

Eclass trained several independent machine learning algorithms (see Table 2) 1000 times in 150 s compared to the CNN model, which trained 10 times in 52 h on an Nvidia Quadro M5000 GPU. The final Eclass risk score for each lesion was the median risk score produced by the eight independent machine learning algorithms. Figure 6 shows the original analysis on the published data along with the performance of the various dermoscopy algorithms used in medical practice, which are abbreviated in the legend and numbered with their respective literature references (see Table 3). CNN was computationally intensive, taking more time to learn diagnostically relevant features than the time required by Eclass. This comparison does not include the computational time required to calculate the imaging biomarkers that fed Eclass, which was about 3 h.

**Fig. 8:**
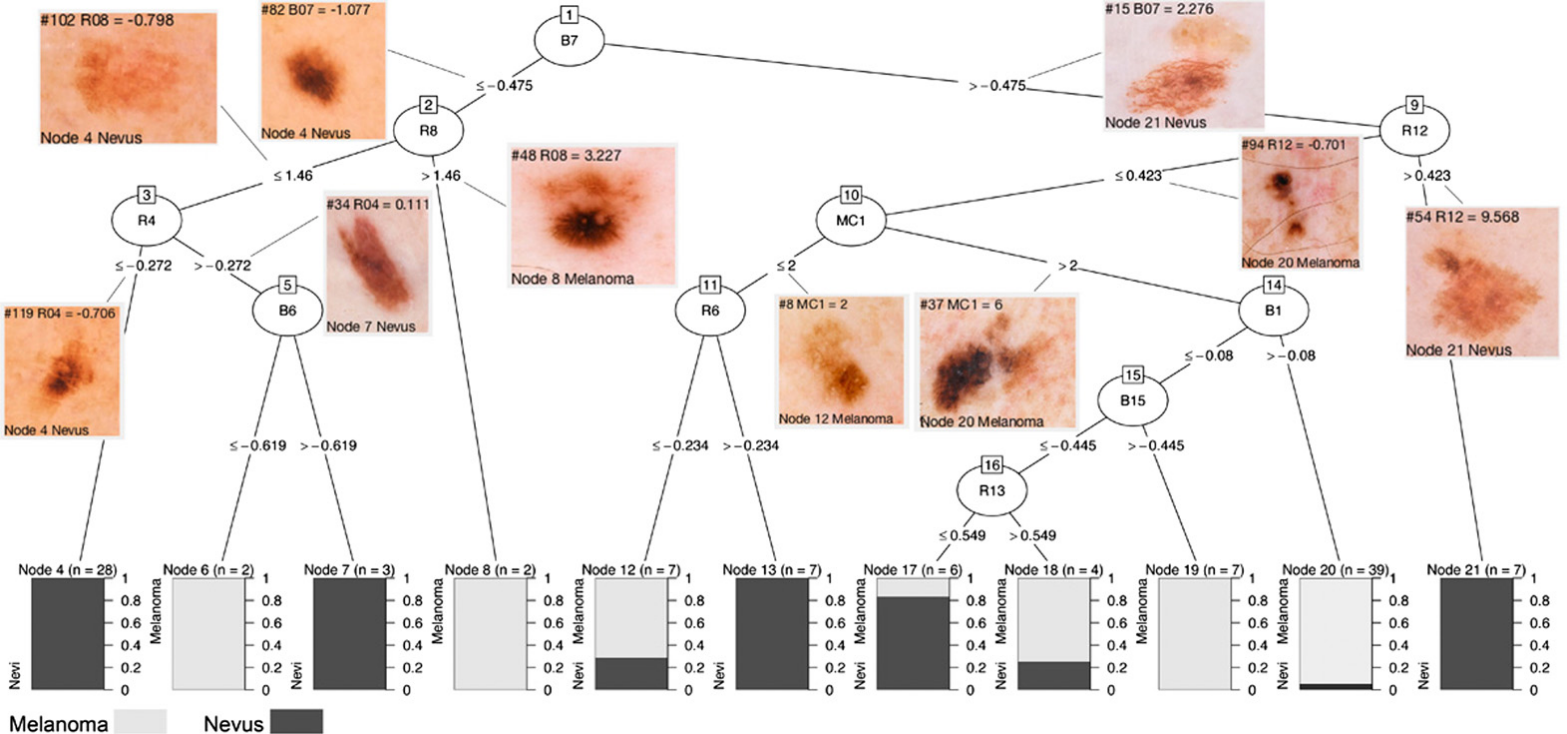
Decision tree built with the C5.0 algorithm. The algorithm was applied to predict lesions type (melanoma vs. nevus) with the full originally published data set that included 112 lesions and 33 IBCs. The decision tree has 10 decision nodes (#1, #2, #3, #5, #9, #10, #11, #14, #15, and #16) and 11 terminal nodes (#4, #6, #7, #8, #12, #13, #17, #18, #19, #20, and #21). The algorithm selected decision nodes based on four IBCs from the blue channel (B1, B6, B7, and B15), five IBCs from the red channel (R4, R6, R8, R12, and R13) and one multicolor IBC (MC1). At the terminal nodes the proportion of melanomas (light gray) and nevi (dark gray) are shown with stacked bar plots. The final classification has yielded 7 pure terminal nodes (#4, #6, #7, #8, #13, #19, and #21) where melanoma or nevi have 100% prevalence. The nodes #4 and #20 together have 59.8% of the lesions and they perfectly discriminate nevi and melanoma, respectively.

**Table 3:** Diagnostic sensitivity and specificity for melanoma detection by human pattern recognition (*) and by machine-augmented pattern recognition (**). The final two listed other techniques (***) represent the current state of commercially available clinical machine-vision systems.

Method	Sensitivity (%)	Specificity (%)
*GP referral†^32^	51	71
*GP referral, dermoscopy†^32^	79	72
*Pattern analysis^33^	85	79
*ABCD^33^	84	75
*ABCD†^34^	56	92
*7-Point checklist^35^	78	65
*7-Point checklist‡^34^	58	92
*CASH^36^	98	68
*Menzies^35^	85	85
*Malignancy grading‡^34^	73	81
*Total body photography^37^	75	74
*Dermoscopy^38^	90	90
*Confocal microscopy^39^	90	86
**Prelim. SIAscopy^40^	50	84
**Prelim. SIAscopy^40^	44	95
**Solar Scan^41^	91	68
**Prelim. Melafind 1^42^	98	44
**Prelim. Melafind 2^42^	91	38
**Image processing^43^	77	87
***SIAscopy^44^	86	65
***Melafind^45^	98	10
Eclass^10^	98	36

† = referral to a dermatologist by a general practitioner nonexpert dermoscopist

‡ = averaged over *in situ* and stage I melanoma.

Performance on the current data set (Validation Set 1) was characterized as the mean and standard deviation of the AUROC. For Eclass, the AUROC was 0.71±0.07 with a 95% confidence interval of [0.56 0.85] while the CNN achieved an AUROC of 0.67 with a 95% confidence interval of [0.63 0.71]. In a Monte Carlo simulation that randomly drew ROCs from the 10 CNN ROCs to compare to ROCs randomly drawn from the 1000 Eclass ROCs, the AUROC was greater for Eclass 74.88% of the time. The AUROC for both models is lower than in other studies with larger numbers of more typical nevi. This performance comparison suggests that codifying dermoscopy features into imaging biomarkers distills information from the image, enabling the Eclass model to operate more efficiently than the CNN and without access to the original image pixels.

The diagnostic score was the least favorite feature of clinicians who tested the IBC App. 10 clinicians, aged 26 to 64 years old, scored the App an average of 2.3 out of 4 for utility in their respective clinical settings. Scores ranged from 2 to 4 with the App being favored by younger clinicians (score=−0.037×Age+4.17, $R^{2}=0.41$). Figures 2(b)–2(d) shows screen captures from the App after revisions based on the clinicians’ feedback in the study data. The major result we achieved with our human subjects’ research on dermatologists is that the dermatologists have a very small bandwidth to process IBCs compared to the analytical bandwidth of the computer. Therefore, we devised a scheme by which we showed only diagnostically significant imaging biomarkers (ones that were very low indicating nevus or very high indicating melanoma) as visual aids. Figure 7 shows this approach by which we compared each IBC value to a population of values, which we verified to be normally distributed, to display it in a clinically-appropriate way so as not to distract the App user with too much unimportant detail.

**Fig. 9:**
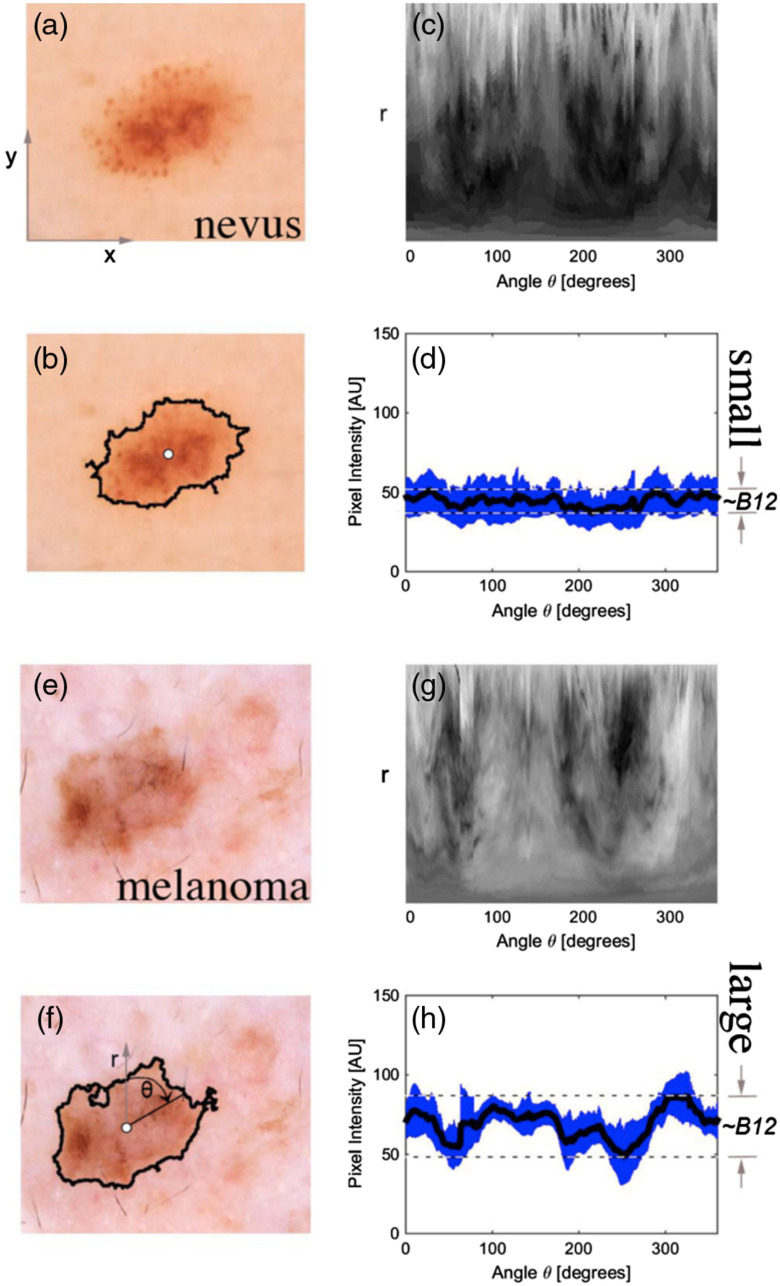
Coordinate transformation and illustration of IBC derivation using angular clock sweep analysis. In images of a nevus (a) and a melanoma (e), lesion border and center (b, f). (c, g) show the blue channel data under a coordinate transformation from x–y to R-θ such that the bottom row of pixels in (g) is the same pixel in f, namely the center pixel (white circle) and the top row of pixels in (g) traces out the lesion border clockwise. (d, h) analyze the pixel brightness statistics (mean in black and standard deviation in blue) of (c, g) in the vertical direction which is along the radial in (b, f). In (d, h), IBC B12, for example, is derived from the radial variation range, which is the vertical separation of the horizontal dashed lines (d, h).

## 4 Discussion

There is an ongoing need to develop digital imaging biomarker libraries in melanoma detection and other diagnostic fields. In contrast to deep learning, which has a convolutional aspect and many features that are hard for clinicians to understand, pre-existing image analytical frameworks (e.g., dermoscopy) can be codified to provide a transparent link between machine and human intelligence. The App we provide^31^ is an example for demonstration that visualizes a subset of the imaging biomarkers on random images drawn from a previously published^10^ study set, highlighting the biomarkers red if they are statistically malignant or in green if they are statistically benign, as defined as falling 1.5 standard deviations above or below the mean value for that imaging biomarker across a reference set of training images, respectively. App users can thus be directed to imaging biomarker visual sensory cues [Fig. 2(c)] of particular diagnostic importance to form a mental analysis before selecting “show diagnostic” to show [Fig. 2(d)] both the CNN and Eclass risk scores as additional inputs in the cue integration process. After making a test diagnosis, selecting “show diagnosis” reveals [Fig. 2(e)] the gold standard biopsy-based diagnosis.

Though we report the median melanoma likelihood produced by the various machine learning approaches as the Eclass score, one approach (the C5.0 decision tree approach) outperformed the Eclass score at 98% sensitivity, yielding sensitivity/specificity = 98%/44% in our originally published study. This result was produced using branching logic. Figure 8 shows an illustration of this branch choice approach, which may be the most promising approach as instructive to visual examination. This analysis may be able to “teach back” to dermatologists both new visual dermoscopic features and new ways to combine IBC evaluations sequentially. Our full diagnostic, which involves calculating a melanoma probability for each machine learning approach and taking the median of those probabilities as the Eclass, may be reduced for translation to visual screening. Thus, automated, unconstrained visual-aided screening with optimized decision trees shows feasibility for human use without computer vision. Also, it may be desirable from the point of view of decreasing computation time and/or complexity during evaluation to reduce the number of statistical classification approaches from the 13 used here to 1 or 2 (e.g., the C5.0 tree in Fig. 8) in cases where such a small subset continues to outperform the Eclass ensemble approach that uses the median result of all the classifiers.

**Fig. 10:**
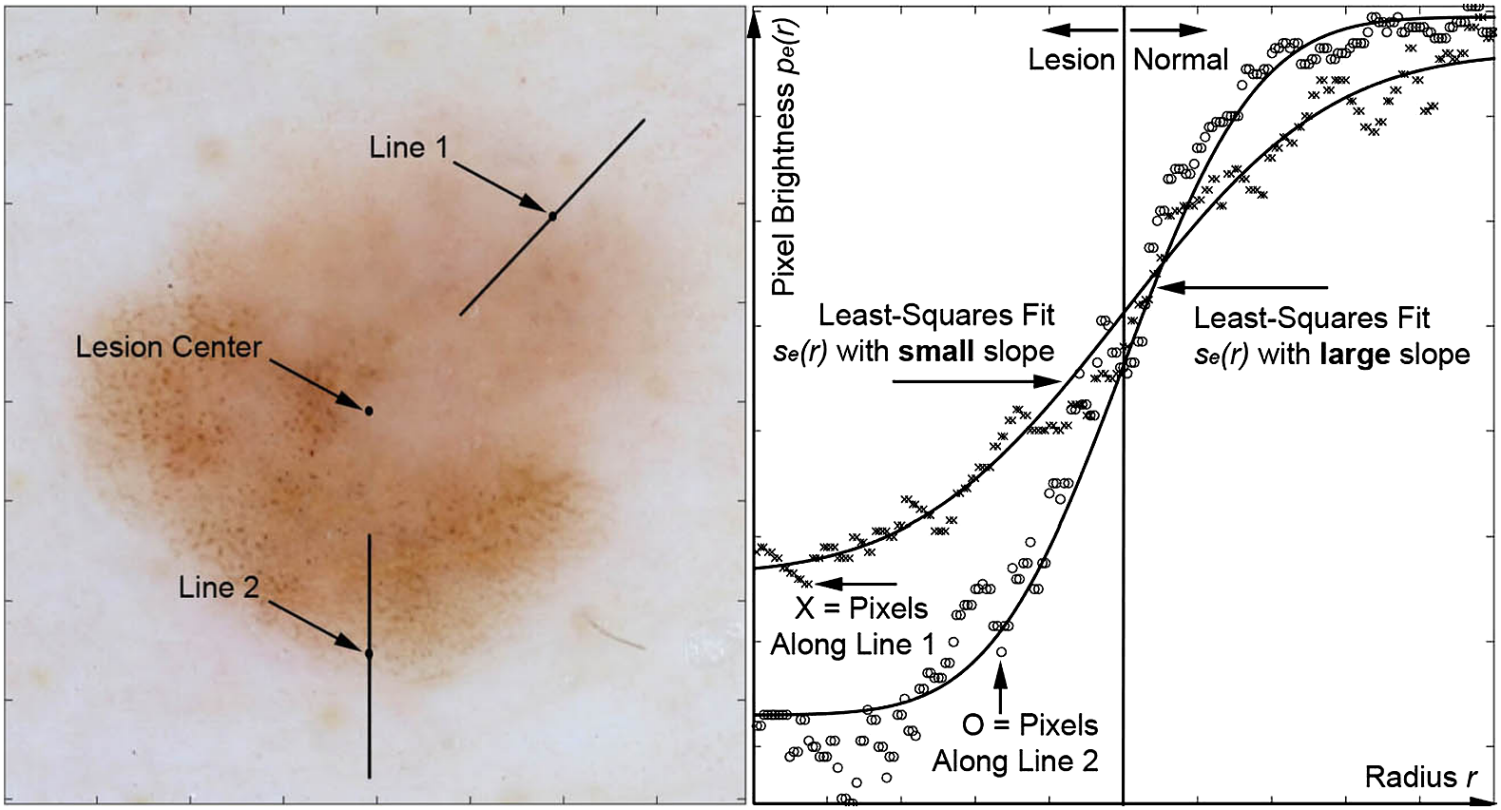
Fitting for edge demarcation. Edge demarcation was quantified as the slope of the transitioning from dark pixels inside the lesion to bright pixels outside the lesion. Increased slope of the fitting mathematical function resulted from increased lesion border demarcation. The two radial lines (Line 1, Line 2) drawn on the lesion include the lesion border from inside the lesion where the pixels are dark inside the lesion to outside the lesion where the pixels are bright in normal skin indicate illustrate two locations where the demarcation is gradual (Line 1) and sharp (Line 2). The pixel brightness extracted along these two lines (x for Line 1 and o for Line 2), $p_{e}\;(r)$ was fit to a mathematical model), $s_{e}\;(r)$ to yield the fitting parameters, which were used to produce IBCs B3, B4, B9, B13, B14, R1, R5, and R10. This includes the edge demarcation slope, which is the slope of the solid line at the lesion border between normal skin and lesion and the error in the fit, which is the sum of the squared differences between the data points, $p_{e}\;(r)$ and the error function fit (solid line) $s_{e}\;(r)$. Melanomas had a sharper border, a higher degree in variability of border sharpness and a greater fitting error.

### 4.1 Analysis in Context

Our findings have implications for frequent machine learning scenarios where the available training set is too small to train high performing deep learning models. Although deep learning systems for breast cancer^46^ screening and melanoma screening^3^ have surpassed human experts in narrow prediction tasks, they have relied on tens or hundreds of thousands of training examples, respectively. Our EClass model, on the other hand, has a model parameter size to data size ratio in the underparameterized “classical” regime of deep learning allowing it to outperform the deep learning model.^47^ Our work provides a head-to-head comparison of CNN versus Eclass on a limited data set and our AUROC of 0.71 is less than the 0.91 published^3^ for melanoma detection based on different training data. Eclass must be evaluated head-to-head against other methods in larger studies in the future, but what makes the present study significant is that the Eclass model was trained on 10× more training images (349) than the number of imaging biomarkers^17^ used as free parameters (349 >10×30). By comparison, the number of CNN model free parameters was ∼20 million. This means that the utilization of imaging biomarkers was a preprocessing step in Eclass (but not CNN) that distilled diagnostic image content. In future work, EClass should be compared to CNN for larger data sets in which the training data size results in an overparameterized EClass model. Although required computational resources were minimal for CNN training on this small dataset, increasing the size of the dataset to take advantage of the high-parameter CNN would significantly increase CNN training resources. For example, increasing the data set size from the current 349 images to the approximately 130,000 images used by Esteva et al.^3^ would result in a significant increase in training cost.

### 4.2 Spectral Properties of IBCs

IBCs generally show that melanoma exhibits spectral and structural irregularity versus benign nevi. This can occur at increasing depth in the skin and be shown in the blue, green, and red channels. Not surprisingly, IBCs exhibit spectrally variant diagnostic statistical significance, and we expect that result to play out in our ongoing hyperspectral imaging study.^48^

One hypothetical mechanism for the spectral dependence of diagnostic importance as a function of wavelength is deeper penetration by longer wavelengths (e.g., red), and thus, the ability to differentially visualize differing three-dimensional tissue or chromophore characteristics of melanoma invading the dermis and superficial epidermal imaging by the shorter (e.g., blue) wavelengths of basal layer atypia or junctional nests of melanocytes associated with melanoma. While more IBCs are needed to cover more clinical presentations, IBCs also still need to be related to underlying tissue structure, including proliferative and invasion patterns of melanoma cells, and molecular pathways impacting pigment distribution. In the green channel, where saturation/desaturation of metabolically active areas of active tumor growth impacts the image, contrast captures polymorphic vasculature associated with melanoma and other skin cancers (basal cell and squamous cell carcinoma).

Within a hyperspectral image of a pigmented lesion, one can include measures of hemoglobin saturation and desaturation, which may help to identify metabolically active regions within lesions. The basis for the “steeper edge slope” (IBC R5) in melanomas does not yet have a cellular basis, but we speculate that it might represent growth of melanocytes in nests at the dermal-epidermal junction at the edge of a melanoma, whereas atypical nevi tend to have only individual junctional melanocytes (nevus cells) that are decreasing in number at the edge of this kind of lesion, whereas deeper nests of melanocytes/nevus cells are organized in the central or “body” region of an atypical nevus.^49^

### 4.3 Strengths and Limitations

A limitation of Eclass using imaging biomarkers in this study was that there were 319 discarded images. At present, our method requires analysis of images that show the complete lesion with full borders and some adjacent normal skin, and the images cannot include hair, markings on the skin, or bubbles in the immersion media optically coupling the imaging device to the skin. In these “defective” images, one or more imaging biomarkers did not successfully compute and Eclass analysis failed but CNN analysis still worked. A number of potential solutions for this exclusion problem could enable practical clinical use, including indications to re-image and remove hair over the lesion. Although this problem must be reduced, clinicians already operate by the “when in doubt, cut it out” rule, so providing that exclusions can be reduced to <10%, we expect clinical utility. A second limitation of the current approach is that it needs retraining for each population (e.g. New York versus Barcelona in this report), so any best future case for the machine learning needs to be indicated for the population on which it was trained.

Since the number of imaging biomarkers is much less than the number of features created by CNNs, the incremental processing cost of added imaging biomarkers is small compared to the overall processing cost difference between Eclass and CNN. A strength of machine learning diagnostics using Eclass and IBCs (that may be challenging from the regulatory perspective) is that the approach can be developed by collaborating researchers combining complementary IBC sets. A repository of executable MATLAB^®^ functions (for IBCs) is needed. Another need is for the exploration of compound models. For instance, the CNN risk score itself could be added as an additional single imaging biomarker in the Eclass approach or even downstream as an added member of the ensemble. The cost would be computational, and the benefit would be that the deep learning could produce images of similar lesions with known diagnoses and similar deep learning fingerprints to which a clinician could confirm visual similarity and thus infer probable diagnosis similarity.

### 4.4 Future Directions

Needed are imaging biomarkers that recognize and correct for image defects to reduce necessary exclusions (a potential bias). This will ensure that more imaging biomarkers compute successfully, potentially leading to diagnostic confidence that the lesion contains only known features and those features have been successfully analyzed. Thus, defects in images that prevent Eclass analysis may be automatically identified and corrected, whether implementing deep learning or not. In contrast, defects in imaging biomarkers present opportunities to improve pathological interpretation of light-tissue interactions and defects in machine learning algorithms arise from biases in training data.

Although Eclass and imaging biomarkers aren’t sufficiently generalized (i.e., Fig. 4 differences across sets), which would be required to analyze lesions in real time from mobile phone-coupled dermatoscopes, Fig. 2 and the App illustrates how the data pipeline from dermatoscope image to guided biopsy decisions could work. This simulated clinical workflow highlights the translational perspective that the dermatologist can be presented a manageable amount of machine learning-augmented reality to make a decision based in part by that input but also by their medical discretion.

## 5 Appendix: : Mathematical Formulas for Melanoma Imaging Biomarker Cues

### 5.1 Multi-color IBCs

Multicolor IBCs were those derived from all the color channels of the red/green/blue image channels simultaneously, as opposed to single-channel IBCs [Eqs. (7)–(36)] presented later that were calculated from individual color channels. Thus, multicolor IBCs only had one version whereas each single color-IBC had three versions (one for each color channel).

Dermoscopy includes analysis of the colors present in any given lesion and there are six colors that dermatologists generally identify in lesions [light brown, dark brown, black, red, blue-gray, and white]. As direct examples, segments of dermoscopic colors were hand-segmented within the published data^10^ set blind to the gold standard diagnoses. At least three images containing segments of each dermoscopic color were chosen for pixel extraction and storage as exemplary for each of the six dermoscopic colors of accepted dermoscopy practice. We generated a simple color metric to assign pixels a categorical color: if the pixel ratio of red to blue was within one standard deviation of the mean for that color, and the same was true for red to green, and blue to green, then the pixel was assigned that color. For each pixel, a sequential check was made for the presence of colors in the order (light brown, dark brown, black, red, blue-gray, and white). In this manner, the two most common colors (light brown, dark brown), were first identified and labeled as the least suspicious group. Next, black and red were identified and labeled (as more suspicious) using the same color identification logic. Finally, blue-gray and white were identified as most suspicious. Thus, the algorithm checked each pixel for each color, leaving the color automatically detected as the last checked (most suspicious) color for that pixel.

A color list (CL) was produced for each lesion indicating the presence or absence of each color. For instance, CL = [1 1 1 0 0] would result from a dermoscopic image where the lesion contained light brown, dark brown, and red but no black or blue-gray/white. MC1 is then the number of dermoscopic colors identified in the lesion.

$$\mathrm{MC}1=\sum_{i=1}^{5}\mathrm{CL}(i).$$ (1)

Let L(y,x) denote an image mask of the lesion segment with value 1 inside the lesion and value 0 outside the lesion. Let $L_{\text{red}}(y,x)$, $L_{\text{green}}(y,x)$, and $L_{\text{blue}}(y,x)$ be masks derived from the red, green, and blue channels of the color image, respectively. MC2 is then the normalized difference in lesion size between the red and blue color channels

$$\mathrm{MC}2=\frac{\sum_{x=1}^{Nx}\sum_{y=1}^{Ny}L_{\text{red}}(y,x)-\sum_{x=1}^{Nx}\sum_{y=1}^{Ny}L_{\text{blue}}(y,x)}{\sum_{x=1}^{Nx}\sum_{y=1}^{Ny}L_{\text{red}}(y,x)}.$$ (2)

Let R(θ) be the length of the radial between the geometric center of the lesion and a point on the lesion border that sweeps over the angle θ from θ=0 to θ=2π radians. Let $R_{R}(\theta )$, $R_{G}(\theta )$, and $R_{B}(\theta )$ be three versions where the geometric centers and the borders are those extracted from $L_{\text{red}}(y,x)$, $L_{\text{green}}(y,x)$, and $L_{\text{blue}}(y,x)$, respectively.

$$R_{\mathrm{var}}(\theta )=\frac{\sigma (R_{R}(\theta ),R_{G}(\theta ),R_{B}(\theta ))}{\left\langle R_{R}(\theta ),R_{G}(\theta ),R_{B}(\theta )\right\rangle }.$$ (3)

MC3 is then the mean coefficient of variation of lesion radii among the color channels, where ⟨ ⟩ denotes the expectation value or mean operator.

$$\mathrm{MC}3={\left\langle R_{\mathrm{var}}(\theta )\rangle \right|}_{\theta =0}^{\theta =2\pi },$$ (4)

where, as an illustration of the definition of the mean value, for a set x that contains n elements

$$\langle x\rangle =\frac{\sum_{i=1}^{n}x_{i}}{n}.$$ (5)

MC4 is the binary presence of blue-gray or white in the image. Figure 3 shows MC4, which is likely the simplest IBC to visualize.

MC4=CL(5). (6)

### 5.2 IBCs with blue-channel diagnostic significance

A large set of IBCs were created based on our angular sweep analysis, shown in Fig. 9. We quantified brightness variation on an angular sweeping arm that connected the geometric center of the lesion and a point on the border tracing that border clockwise. From the center, radial arms projected to the lesion border and rotating clockwise were used as regions of interest to quantify image characteristics along the arc of rotation. The series of arcs created by radial sweep around the center covering the entire 360-degree view of the lesion, was analogous to the sweep of hands around an analog clock. The IBC-producing mathematical operations (given in Sec. S5 in the Supplementary Materials^13^ for Ref. 10) either produced direct transformations of the actual data (i.e., Fig. 9) or quantified differences between the data and mathematical models used to estimate the data’s deviation from smoothly transitioning functions (i.e., Fig. 10).

Let $p(r_{1})$ be the pixel brightness along a radial line $r_{1}$ connecting the center point of the lesion and a point on the peripheral edge of the lesion. Let $R_{m}(\theta )$ be the mean pixel brightness $\left\langle p(r_{1})\right\rangle$ along a set of lines that vary as specified by the angle θ. As θ varies in increments of dθ one full rotation from zero to 2π radians (360 degrees), the set of lines $r_{1}$ sweep the lesion like a clock arm sweeping an analog clock.

$$R_{m}(\theta )={\left\langle p(r_{1})\rangle \right|}_{\theta =0}^{\theta =2\pi },$$ (7)

$$R_{std}(\theta )={\sigma (p(r_{1}))|}_{\theta =0}^{\theta =2\pi },$$ (8)

where an illustration of the definition of the standard deviation, for a set x that contains n elements

$$\sigma (x)=(\frac{1}{n-1}\overset{i=n}{\underset{i=1}{\sum }}{\left(x_{i}-\langle x\rangle \right)}^{2}).$$ (9)

B1 is then the average of the absolute value of the derivative of $R_{m}(\theta )$ over the angular sweep is the mean instantaneous brightness shift from one angular analysis position to the next over the entire 360-degree angular range.

$$B1={\left\langle (|R_{m}(\theta_{n})-R_{m}(\theta_{n+1})|)\rangle \right|}_{\theta =0}^{\theta =2\pi }.$$ (10)

B2 is the variance over the angular sweep of the variance in pixel brightness over the radial sampling arm. This variable is increased when there are some angles at which the lesion contains even pigmentation but others that contain variable pigmentation such as in reticular or globular patterns of bright and dark areas.

$$B2={\sigma (R_{\mathrm{std}}(\theta ))|}_{\theta =0}^{\theta =2\pi }.$$ (11)

Let $p_{e}(r_{2})$ be the pixel brightness along a second radial line $r_{2}$ of the same length as $r_{2}$ and at the same angular sweep angle θ but extending from half-to-1.5 times the lesions radius R(θ) instead of 0-to-1 such as to be centered on the border between lesion and normal skin. $P_{e}(r)$ has the characteristic that half of its pixels (within the lesion) are darker than the other half of its pixels (outside the lesion). Let $s_{e}$® be a mathematical model error function across the lesion border with three fitting parameters: Min, Max, and Slope that are iteratively adjusted to minimize the least squares difference between $p_{e}(r)$, the data and $s_{e}(r)$. erf(x) is defined as twice the integral of the Gaussian distribution with 0 mean and variance of 1/2, as shown below with the dummy variable t. Considering $r_{b}$ as the lesion border pixel with approximately the mean pixel brightness in $p_{e}(r)$ and exactly the mean brightness of $s_{e}(r)$, $s_{e}(r)$ is defined as

$$\mathrm{erf}(x)=\frac{2}{\sqrt{\pi }}\overset{x}{\underset{0}{\int }}e^{-t^{2}}\mathrm{dt},$$ (12a)

$$f_{e}(r)=\frac{\mathrm{erf}(\frac{r-r_{b}}{\text{Slope}})}{2},$$ (12b)

$$s_{e}(r)=\mathrm{Min}+\{f_{e}(r)-\min[f_{e}(r)]\}\times \mathrm{Max}.$$ (12c)

B3 is then the mean error between the model $s_{e}(r)$ and the data $p_{e}(r)$ evaluated over a range equal to the distance between the center and the lesion border but centered on the edge of the lesion. This error measurement is high if the lesion brightness does smoothly transition between dark inside the lesion and bright outside the lesion. The fitting algorithm, fminsearch() in MATLAB^®^ (Mathworks Inc., Natick, Massachusetts), was limited to 200 fitting iterations. If convergence was reached before the 200-iteration limit, the result was flagged as one type while fits that were cut off at the 200-iteration limit were flagged as a second type. B3 included only results of the second type, that did not converge by the time the iteration limit was reached.

$$B3={\left\langle \overset{R=1.5D}{\underset{R=0.5D}{\sum }}{\left[p_{e}(r)-s_{e}(r)\right]}^{2}\rangle \right|}_{\theta =0}^{\theta =2\pi }.$$ (13)

B4 is the mode error, calculated the same as B3 but with the mode() operator instead of the mean ⟨ ⟩ operator, calculated for only the data that exceeded the number (200) of fitting iterations allowed.

$$B4=\text{mode}{\left(\overset{R=1.5D}{\underset{R=0.5D}{\sum }}{\left(p_{e}(r)-\mathrm{erf}(r)\right)}^{2})\right|}_{\theta =0}^{\theta =2\pi }.$$ (14)

B5 is the standard deviation of the set of derivative values of the mean brightness. The variance of the derivative of brightness describes how much variability in the instantaneous change in brightness there is over the angular sweep. If some angular ranges are flat (low intra-range brightness derivative) and some ranges vary wildly, this variable will have a high value.

$$B5=\sigma (\frac{dR_{m}}{d\theta })={\sigma (|R_{m}(\theta_{n})-R_{m}(\theta_{n+1})|)|}_{\theta =0}^{\theta =2\pi }.$$ (15)

B6 was calculated like B3 except that it used all data and was not restricted to the data requiring more fitting iterations than MATLAB^®^ was allowed to execute. Similarly, B7 used only the fits that did not require more iterations than (200) the maximum number of fitting iterations allowed.

A watershed analysis was developed to identify pigmented network branches. First, gray-scale images extracted from individual channels were passed through a rank filter which reset the gray-scale value of each pixel to the rank in brightness of the pixel under consideration with its group of neighboring pixels. This step was needed prior to the watershed analysis to act as a high-pass spatial filter and eliminate overall brightness variations in the lesion, leaving the local variations such as those caused by pigmented networks to be identified by the watershed analysis. Branches, which were skeletonized to a single pixel width down their spine, were characterized by three features: their length, their mean brightness, and their angle with respect to the lesion centroid. The MR clock sweep scored the mean pixel intensity of the branches $I_{\text{branch}}(\theta )$, the standard deviation of intrabranch pixel intensity variation $\sigma_{\text{branch}}$, the mean length of the branches $L_{\text{branch}}(\theta )$ and the total number of branches $N_{\text{branch}}(\theta )$ within a differential angle element that traced with the clock MR clock sweep. B8 is then the normalized inter-branch pixel intensity variation.

$$B8=\frac{\sigma ({I_{\text{branch}}(\theta )|}_{\theta =0}^{\theta =2\pi })}{\left\langle {I_{\text{branch}}(\theta )|}_{\theta =0}^{\theta =2\pi }\right\rangle }.$$ (16)

B9 is the standard deviation of the error measurement like in B3, except that the standard deviation operator σ is used instead of the mean ⟨ ⟩ operator. B9 was evaluated only for fits requiring more fitting iterations than the 200 iterations allowed.

$$B9={\sigma \{\overset{R=1.5D}{\underset{R=0.5D}{\sum }}{\left[p_{e}(r)-\mathrm{erf}(r)\right]}^{2}\}|}_{\theta =0}^{\theta =2\pi }.$$ (17)

B10 is the normalized angular coefficient of brightness variation.

$$B10=\frac{\sigma [R_{m}(\theta )]}{\left\langle R_{m}(\theta )\right\rangle }.$$ (18)

B11 The standardized variance of branch lengths.

$$B11=\frac{{\sigma (L_{\text{branch}})|}_{\theta =0}^{\theta =2\pi })}{\left\langle {L_{\text{branch}}|}_{\theta =0}^{\theta =2\pi }\right\rangle }.$$ (19)

B12 is the normalized range of angular brightness.

$$B12=\frac{\max[R_{m}(\theta )]-\min[R_{m}(\theta )]}{\left\langle R_{m}(\theta )\right\rangle }.$$ (20)

B13 is calculated as is B6 except the standard deviation operator σ is used instead of the mean ⟨ ⟩ operator. Like B6, B13 used all the data.

$$B13={\sigma (\overset{R=1.5D}{\underset{R=0.5D}{\sum }}{\left[p_{e}(r)-\mathrm{erf}(r)\right]}^{2})|}_{\theta =0}^{\theta =2\pi }.$$ (21)

B14 Is the standard deviation σ() of the error measurement as in B13 except that B14 was evaluated only for the fits that completed within the allowed number (200) of fitting iterations.

B15 Is the mean intrabranch coefficient of variation.

$$B15=\langle {\frac{\sigma (I_{\text{branch}}(\theta ))}{\left\langle I_{\text{branch}}(\theta )\right\rangle }|}_{\theta =0}^{\theta =2\pi }\rangle .$$ (22)

### 5.3 IBCs with green-channel diagnostic significance

Let ${\text{Perim}}_{G}$ be the length of the perimeter of the lesion segment in the green channel $L_{\text{green}}$. G1 is the length of the lesion segment border normalized by the square root of the area of the lesion segment.

$$G1=\frac{{\text{Perim}}_{G}}{\sqrt{\sum_{x=1}^{Nx}\sum_{y=1}^{Ny}L_{\text{green}}}}-\frac{2\pi }{\sqrt{\pi }}.$$ (23)

### 5.4 IBCs with red-channel diagnostic significance

The fitting algorithm yielded a slope S for the sigmoidal edge fit. R1 was the standard deviation of the slope fit values

$$R1={\sigma (S)|}_{\theta =0}^{\theta =2\pi }.$$ (24)

R2 is the fractal dimension of the lesion segment binary image as defined as^50^

$$R2=D[L_{\text{red}}(y,x)].$$ (25)

Each branch segment in terminated on two ends in either a branch point or an end point. R3 is the connectedness of the pigmented network, defined as the ratio of the number of branch points $N_{\mathrm{BP}}$ to the number of endpoints $N_{\mathrm{EP}}$.

$$R3=\frac{N_{\mathrm{BP}}}{N_{\mathrm{EP}}}.$$ (26)

R4 is the size of the lesion segment $L_{\text{red}}$, which is the sum of the binary mask valued at one inside the lesion segment and zero outside the lesion segment.

$$R4=\overset{Nx}{\underset{x=1}{\sum }}\overset{Ny}{\underset{y=1}{\sum }}L_{\text{red}}.$$ (27)

R5 is the mean slope (S) for the edge fit function $s_{e}(r)$ [as used in Eq. (13)] evaluated only for the fits that did not require more iterations of the fminsearch() operator than the 200 allowed.

$$R5=\langle {S|}_{\theta =0}^{\theta =2\pi }\rangle .$$ (28)

Let the instantaneous radius of the lesion, as in Eq. (3), be denoted by $R_{R}(\theta )$ over the angular sweep of θ. R6 is then the coefficient of variation in the lesion radius over the angular sweep

$$R6=\frac{\sigma (R_{\text{red}}{\left(\theta )\right|}_{\theta =0}^{\theta =2\pi })}{\left\langle {R_{\text{red}}(\theta )|}_{\theta =0}^{\theta =2\pi }\right\rangle }.$$ (29)

Let $N_{b}(\theta ,d\theta )$ be the number of pigmented network branches identified in a differentiual angle emelment dθ as a function of angle θ over the angular sweep. R7 is then the range in number of branches detected as a function of angle.

$$R7=\max[N_{\text{branch}}(\theta ,d\theta )]-\min[N_{\text{branch}}(\theta ,d\theta )].$$ (30)

R8 is the range in the standard deviation of pixel brightness on the angular sweep arm over the angular sweep.

$$R8=\max({R_{\mathrm{std}}(\theta )|}_{\theta =0}^{\theta =2\pi })-\min({R_{\mathrm{std}}(\theta )|}_{\theta =0}^{\theta =2\pi }).$$ (31)

Pixels with the lesion segment were scored as a set $P_{\text{lesion}}$. The coefficient of variation for pixels within the lesion segment was calculated by dividing the standard deviation of intralesional pixel brightness by the mean lesional pixel brightness. R9 is then the coefficient of variation in pixel brightness within the lesion.

$$R9=\frac{\sigma (P_{\text{lesion}})}{\left\langle P_{\text{lesion}}\right\rangle }.$$ (32)

R10 is the mode error, calculated the same as B4, but evaluated only for the fits that did not exceed the number of fitting iterations (200) allowed.

$$R10=\text{mod}e{\left(\overset{R=1.5D}{\underset{R=0.5D}{\sum }}{\left[p_{e}(r)-\mathrm{erf}(r)\right]}^{2})\right|}_{\theta =0}^{\theta =2\pi }.$$ (33)

The maximum asymmetry of the lesion was normalized by the eccentricity of the lesion E as calculated using the stats. Ecentricity function in MATLAB^®^. This normalization enabled de-emphasis of uniform ovals as asymmetric. R11 is then the maximum asymetry of the lesion silhouette

$$R11=\max(\frac{A}{E}).$$ (34)

R12 is the sum of the normalized derivitive in lesion radius D over the angular sweep

$$R12=\overset{\theta =2\pi }{\underset{\theta =0}{\sum }}\mathrm{abs}[R_{\text{red}}(\theta ,d\theta )-R_{\text{red}}(\theta -1,d\theta )].$$ (35)

R13 is the asymetry of the lesion silhouette evaluated in the standard technique (Fig. S10 in the Supplementary Material for Ref. 10)^13^

$$R13=A|\theta_{\mathrm{sym}}-\frac{\pi }{2}.$$ (36)
